# Integrated Multi-Trait Analysis of Photosynthetic Carbon Assimilation Strategies in *Dendrobium* Species

**DOI:** 10.3390/plants15203207

**Published:** 2026-10-19

**Authors:** Zhe Yang, Lingzhi Wei, Lihui Peng, Yujing Wei, Zhenhai Deng, Zhongchen Xiong, Xiao Wei, Tao Ding, Shengfeng Chai

**Affiliations:** 1 Key Laboratory of Plant Functional Phytochemicals and Sustainable Utilization, Guangxi Institute of Botany, Guilin 541006, China; yang2000zhe@126.com (Z.Y.);; 2 College of Life Sciences, Hebei University, Baoding 071000, China; 3 Yachang Orchid National Nature Reserve Management Center, Baise 533209, China

**Keywords:** *Dendrobium* species, carbon assimilation, crassulacean acid metabolism, carbon isotope composition, leaf anatomy

## Abstract

*Dendrobium* species are valuable medicinal and ornamental orchids whose epiphytic or lithophytic growth exposes them to fluctuating water availability. Comparative evidence on their carbon-assimilation strategies can help identify physiological traits relevant to conservation and cultivation. We compared nine *Dendrobium* species using diel net CO_2_ exchange, leaf carbon isotope composition (δ^13^C), dawn–dusk titratable acidity differences (ΔH^+^), leaf anatomy, and the mesophyll succulence index (Sm), and evaluated agreement between physiological and structural indicators. *D. parishii*, *D. aphyllum*, and *D. anosmum* showed concordant patterns consistent with substantial CAM-associated carbon assimilation. *D. cariniferum*, *D. gibsonii*, and *D. hancockii* showed predominantly C_3_-associated patterns under the conditions examined. *D. linawianum*, *D. moschatum*, and *D. crystallinum* combined C_3_-like isotope signatures with weak reported nocturnal CO_2_ exchange and variable acidity differences; these observations are described provisionally as CAM-like characteristics and do not establish facultative CAM or definitive C_3_/CAM intermediate status. Leaf thickness and Sm overlapped among physiological groups, limiting their diagnostic value. Exploratory correlations across nine species suggested associations among physiological indicators, but small sample size and unaccounted phylogenetic relatedness limit generalization. This comparative dataset supports complementary physiological measurements when evaluating *Dendrobium* carbon assimilation.

## 1. Introduction

Photosynthesis supplies the carbon required for plant growth, while the organization of carbon fixation influences how plants use light and water [1,2]. C_3_, C_4_, and crassulacean acid metabolism (CAM) differ in their mechanisms of carbon acquisition and concentration [3,4,5]. CAM couples nocturnal fixation of CO_2_ with organic acid storage and subsequent daytime decarboxylation, allowing carbon assimilation with reduced daytime stomatal opening [6,7,8]. The contribution of CAM varies from low levels of nocturnal acid cycling to substantial atmospheric CO_2_ uptake at night [9,10,11]. This variation makes CAM relevant to understanding how plants maintain carbon gain under intermittent water limitation.

*Dendrobium* provides a useful system for examining these strategies because it combines medicinal and ornamental importance with considerable ecological and physiological diversity [12,13,14]. Many species grow on trees or rocks, where access to water fluctuates despite a humid regional climate. Experiments with epiphytic orchids have identified complementary roles for leaf cuticles and pseudobulb water storage in maintaining water balance [15]. Within *Dendrobium*, studies of *D. officinale* and other species have documented contrasting C_3_- and CAM-associated patterns using gas exchange, carbon isotopes, and acidity measurements [16,17]. Comparative chloroplast genomic work has also examined *Dendrobium* species with different photosynthetic patterns [18]. These findings motivate comparisons that link carbon assimilation with structural traits across species cultivated under a common environment.

Recent experiments have extended this physiological perspective to stress responses and their regulation. Xia et al. [19] compared drought responses in three *Dendrobium* species with different photosynthetic pathways and identified species-dependent transcriptional responses, including differences in phosphoenolpyruvate carboxylase (PEPC) expression. Their results connect variation in carbon metabolism with the molecular response to water shortage. In the CAM orchid *Dendrobium* Sonia ‘Earsakul’, drought and heat produced distinct effects on carbon exchange, with combined stress imposing a greater physiological burden [20]. Experimental work on tropical epiphytic orchids further demonstrates that CAM expression can change with environmental conditions [21]. Such studies establish the importance of species-specific responses and provide a basis for selecting contrasting taxa for cultivation and conservation research.

An integrated comparison requires measurements that capture different aspects of carbon assimilation. Gas exchange resolves the timing of net CO_2_ uptake, whereas leaf δ^13^C reflects the contribution of fixation processes during tissue formation [22,23]. Dawn–dusk titratable acidity differences provide complementary evidence of overnight acid accumulation [24]; orchid surveys show why this information is especially useful for identifying weak CAM expression alongside C_3_-like isotope signatures [25]. Leaf anatomy and mesophyll succulence describe the structural context for water and acid storage [26,27]. Recent comparative studies associate CAM with combinations of anatomical traits, although relationships can differ among lineages [28,29]. Here, we compare nine *Dendrobium* species using diel net CO_2_ exchange, δ^13^C, titratable acidity, leaf anatomy, and the mesophyll succulence index (Sm). We aim to characterize their carbon-assimilation patterns, examine agreement among physiological indicators, and assess how structural traits relate to those patterns. The resulting comparison provides a physiological basis for selecting species and traits for subsequent irrigation and light-response studies.

## 2. Results

### 2.1. Diel Patterns of Net CO_2_ Exchange

**Figure 1:**
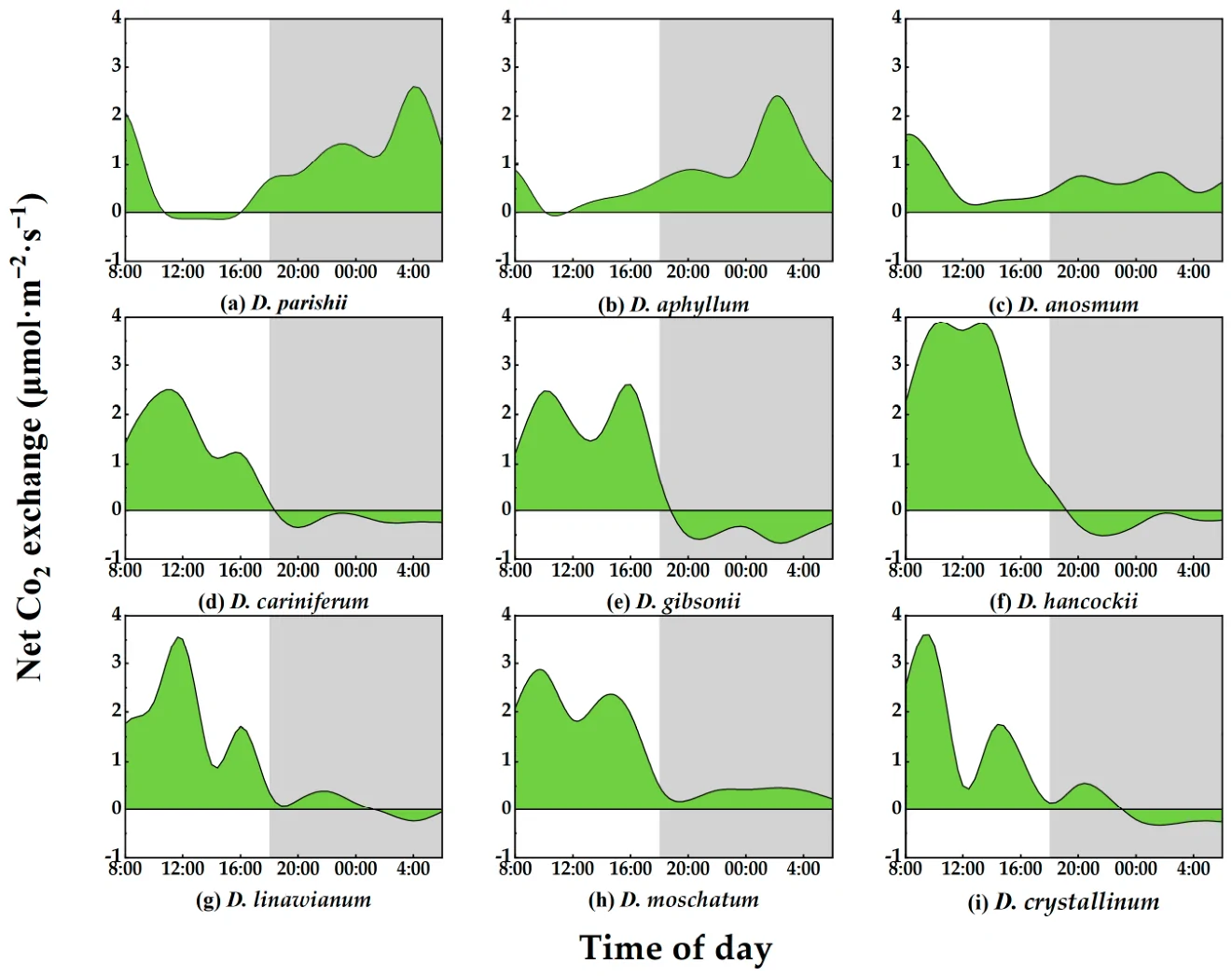
Net CO_2_ exchange in nine *Dendrobium* species at 12 nominal two-hour times from 08:00 on 7 October to 06:00 on 8 October 2023. The elapsed observation window is 22 h. Three independent plants were measured per species. Curves show the reported species means. The shaded interval marks the evening-to-morning period beginning at 18:00; residual light at 18:00 and low light at 06:00 mean that this interval is not uniformly dark. The horizontal line marks zero net exchange.

The nine *Dendrobium* species showed contrasting diel net CO_2_ exchange patterns (Figure 1). *D. cariniferum*, *D. gibsonii* and *D. hancockii* had negative mean exchange at the six nominal 20:00–06:00 sampling times. *D. parishii*, *D. aphyllum* and *D. anosmum* had positive mean exchange at these times, consistent with a greater contribution of nocturnal or early-morning carbon uptake. *D. parishii* and *D. aphyllum* reached their largest sampled values at 04:00 and 02:00, respectively; *D. anosmum* had its largest sampled value at 08:00. *D. linawianum* showed weak positive values at 20:00, 22:00 and 00:00, followed by negative values at 02:00–06:00. *D. crystallinum* showed weak positive values at 20:00 and 22:00, followed by negative values from 00:00 onward. *D. moschatum* had positive reported mean exchange at all 12 sampling times.

### 2.2. Carbon Isotope Composition and Titratable Acidity

**Figure 2:**
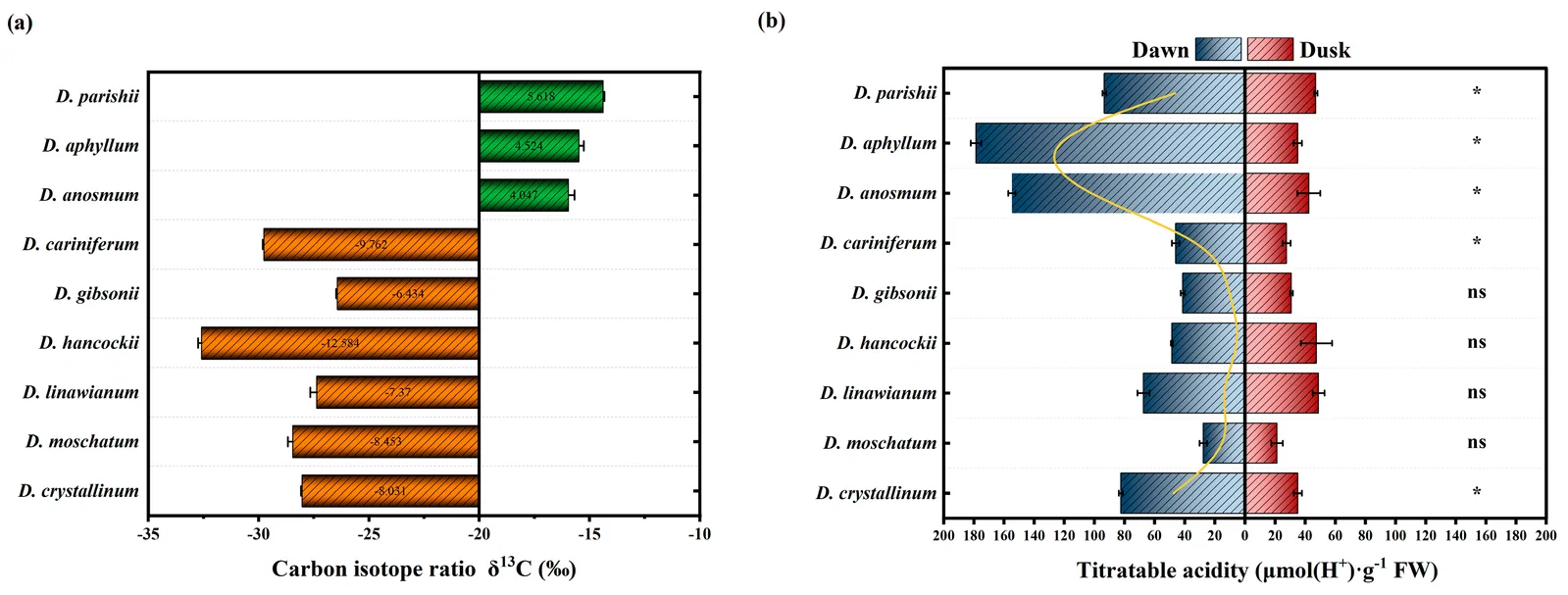
(**a**) Bulk-leaf carbon isotope composition (δ^13^C, ‰). The horizontal axis gives actual δ^13^C values, whereas numbers inside the bars indicate deviations from −20‰, calculated as δ^13^C − (−20‰). The −20‰ line is a descriptive reference. (**b**) Titratable acidity at 06:00 and 18:00, and their difference ΔH^+^, in μmol H^+^ g^−1^ FW. The biological sampling design comprised three independent plants per species. Bars represent means ± SD (*n* = 3 independent plants per time point). Asterisks indicate significant dawn–dusk differences (* *p* < 0.05), and ns indicates no significant difference (*p* ≥ 0.05). The yellow curve represents ΔH^+^, calculated as mean dawn acidity minus mean dusk acidity.

Leaf δ^13^C differed descriptively among species (Figure 2a). *D. parishii*, *D. aphyllum*, and *D. anosmum* had values less negative than −20‰, whereas the other six species had more negative values. These patterns are consistent with contrasting contributions of nocturnal and daytime assimilation during tissue development.

Mean titratable acidity at 06:00 exceeded that at 18:00 in the species-level summary (Figure 2b). The ΔH^+^ means for *D. parishii*, *D. aphyllum* and *D. anosmum* were 46.373, 143.544 and 112.332 μmol H^+^ g^−1^ FW, respectively; *D. aphyllum* had the largest reported difference. *D. crystallinum* also showed a relatively large difference (47.355 μmol H^+^ g^−1^ FW). The remaining species had mean differences below 19 μmol H^+^ g^−1^ FW, with the smallest reported mean in *D. hancockii* (1.088 μmol H^+^ g^−1^ FW).

### 2.3. Leaf Anatomical Structure

**Figure 3:**
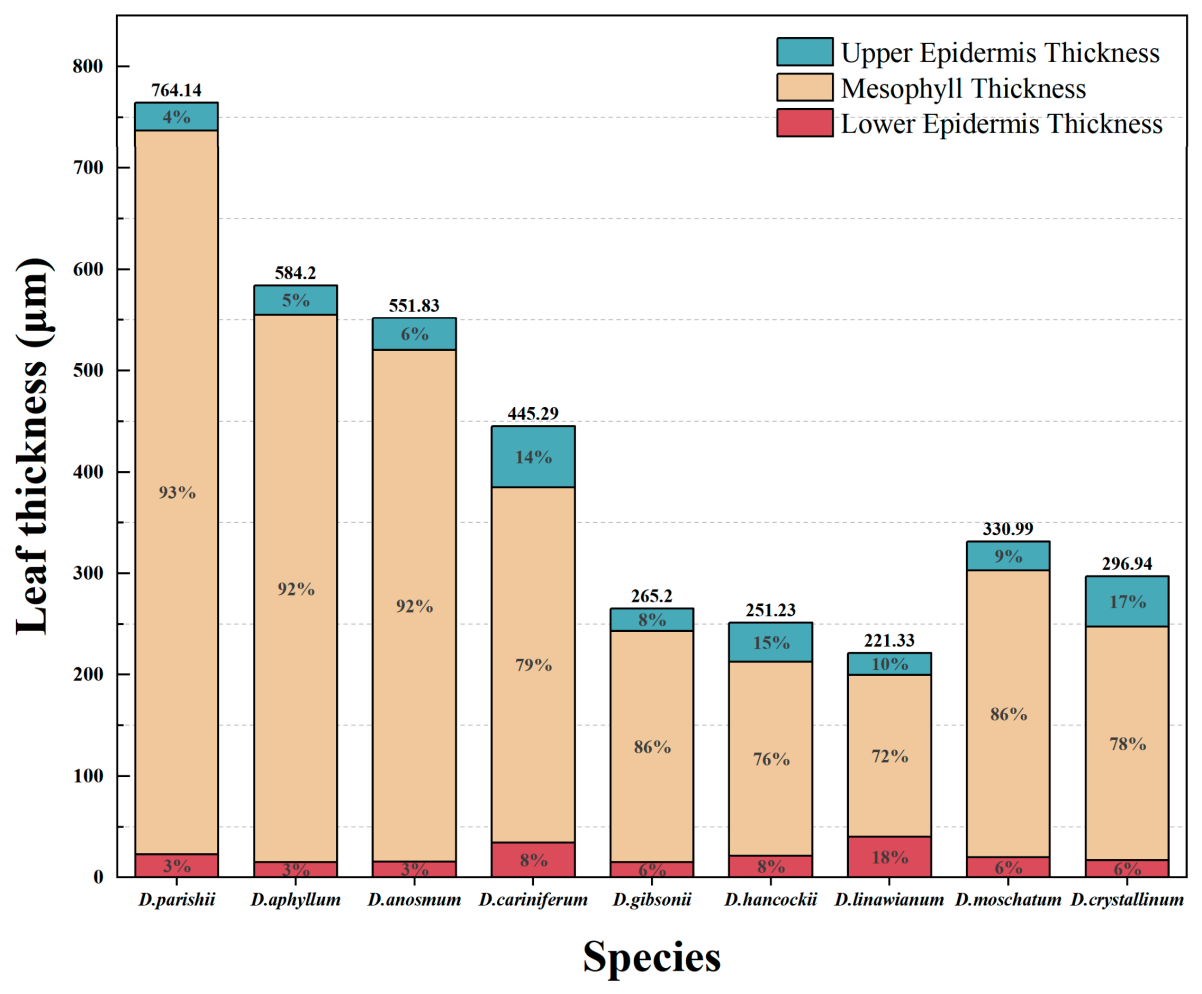
Leaf thickness and tissue composition in nine *Dendrobium* species. The original stacked bars show upper epidermis, mesophyll and lower epidermis thickness (μm); totals are the sums of tissue components.

**Figure 4:**
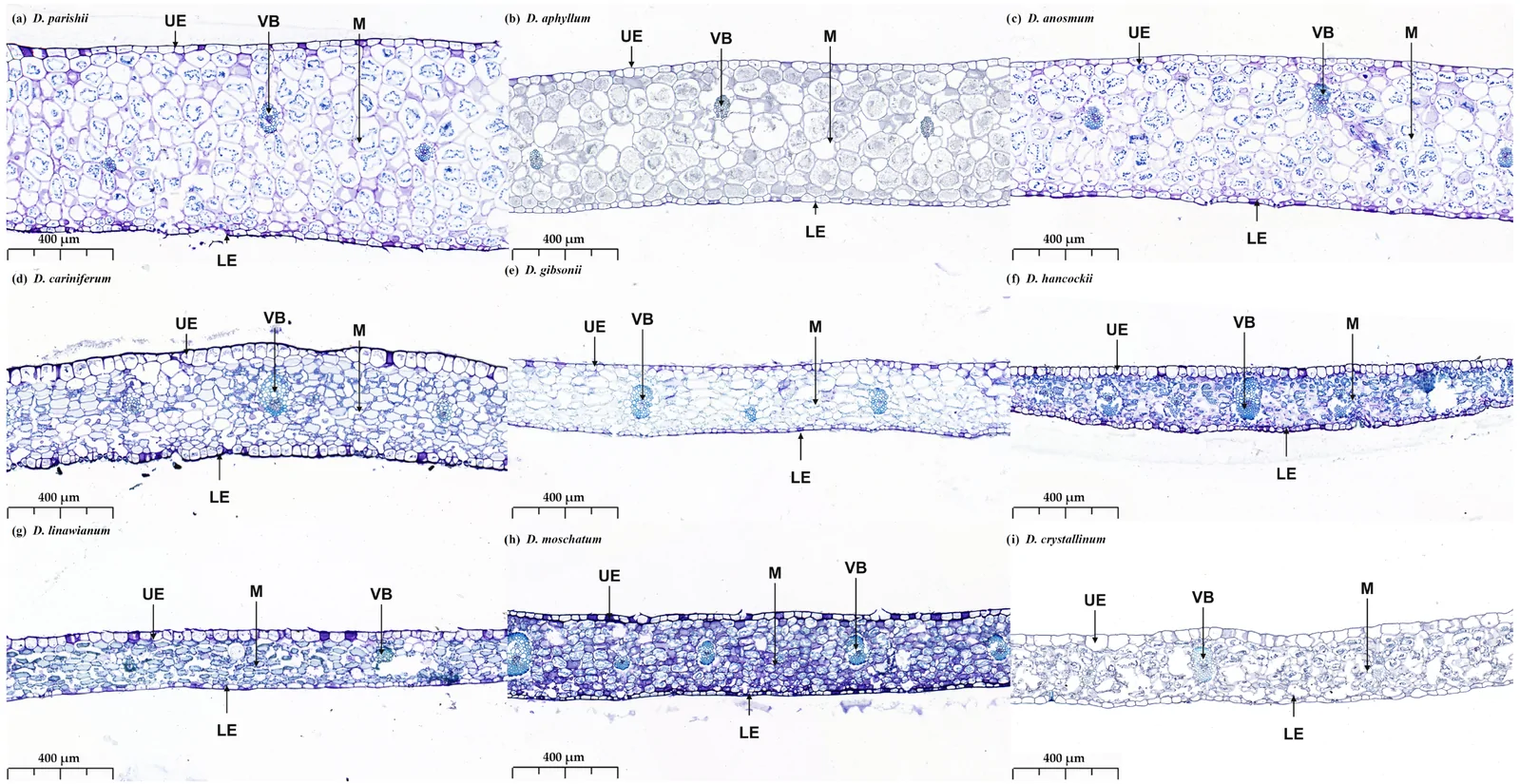
Leaf transverse sections of (**a**) *D. parishii*, (**b**) *D. aphyllum*, (**c**) *D. anosmum*, (**d**) *D. cariniferum*, (**e**) *D. gibsonii*, (**f**) *D. hancockii*, (**g**) *D. linawianum*, (**h**) *D. moschatum*, and (**i**) *D. crystallinum*. Arrows identify UE, upper epidermis; LE, lower epidermis; M, mesophyll; and VB, vascular bundle. Scale bars: 400 μm in each panel.

Leaf thickness and tissue composition are summarized in Figure 3. Leaf sections showed upper and lower epidermal layers, mesophyll and vascular tissues (Figure 4). Mesophyll was not clearly differentiated into palisade and spongy regions in the presented sections. In the updated component-based comparison, total thickness is the sum of upper epidermis, mesophyll and lower epidermis thickness (Figure 3). *D. parishii* had the largest total (764.14 μm), including 714.07 μm of mesophyll. *D. aphyllum* and *D. anosmum* had totals of 584.20 and 551.83 μm, respectively. *D. cariniferum* was also relatively thick (445.29 μm), despite its C_3_-associated physiological pattern. The revised *D. linawianum* entry was the thinnest (221.33 μm).

### 2.4. Mesophyll Succulence Index

**Figure 5:**
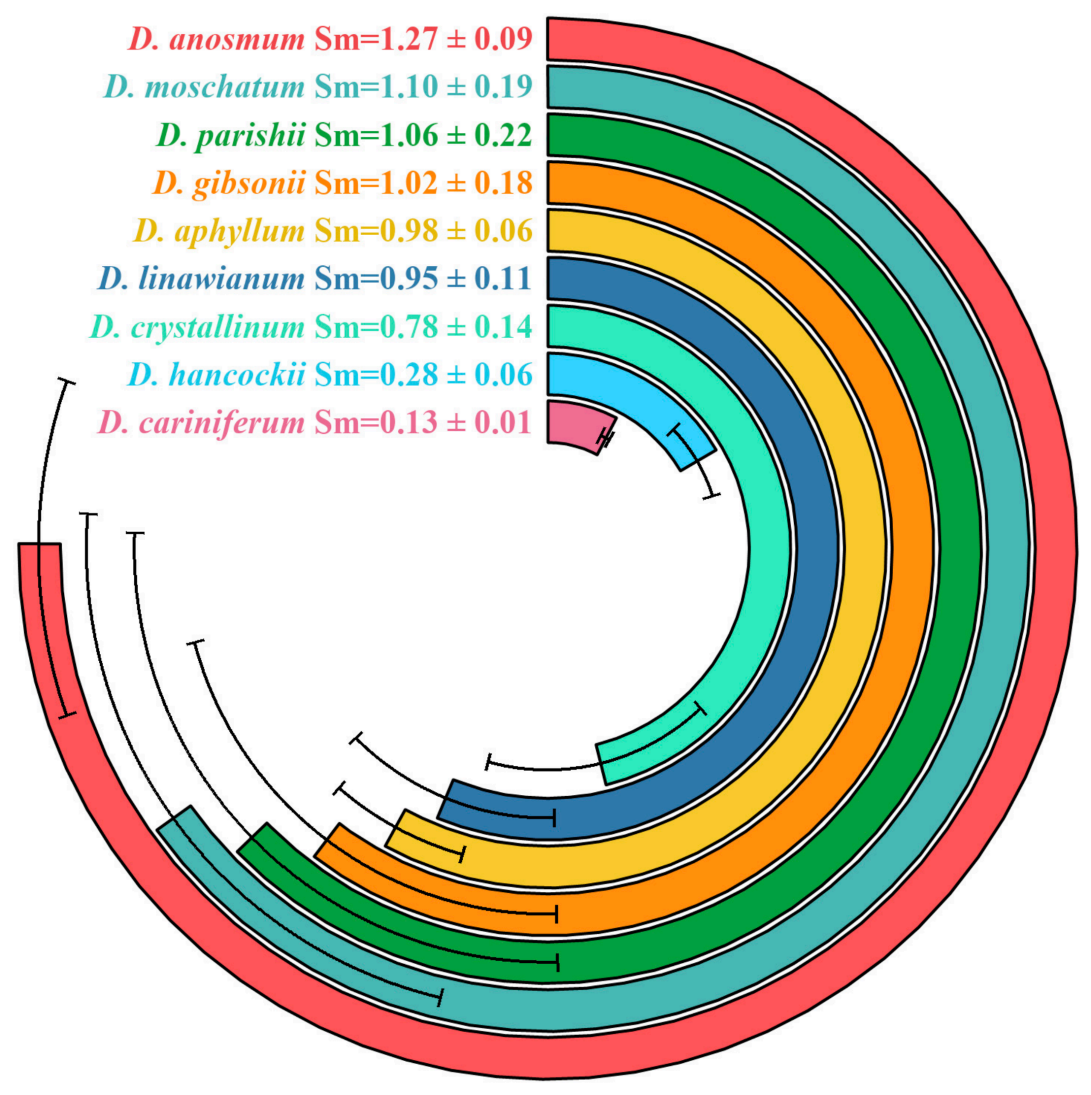
Mesophyll succulence index (Sm, g water mg^−1^ chlorophyll) in nine *Dendrobium* species. Arc lengths represent mean Sm values, and error bars indicate standard deviations (SD) from three independent plants per species (*n* = 3). Values shown beside the species names are means ± SD. Water fraction and chlorophyll content are expressed on a fresh-mass basis, as described in Section 4.6.

Sm varied among species (Figure 5). *D. parishii*, *D. anosmum*, *D. gibsonii* and *D. moschatum* had values of 1.06, 1.27, 1.02 and 1.10 g water mg^−1^ chlorophyll, respectively. *D. aphyllum*, *D. linawianum* and *D. crystallinum* had values of 0.98, 0.95 and 0.78, while *D. cariniferum* and *D. hancockii* had lower values of 0.13 and 0.28.

### 2.5. Correlation Analysis

**Figure 6:**
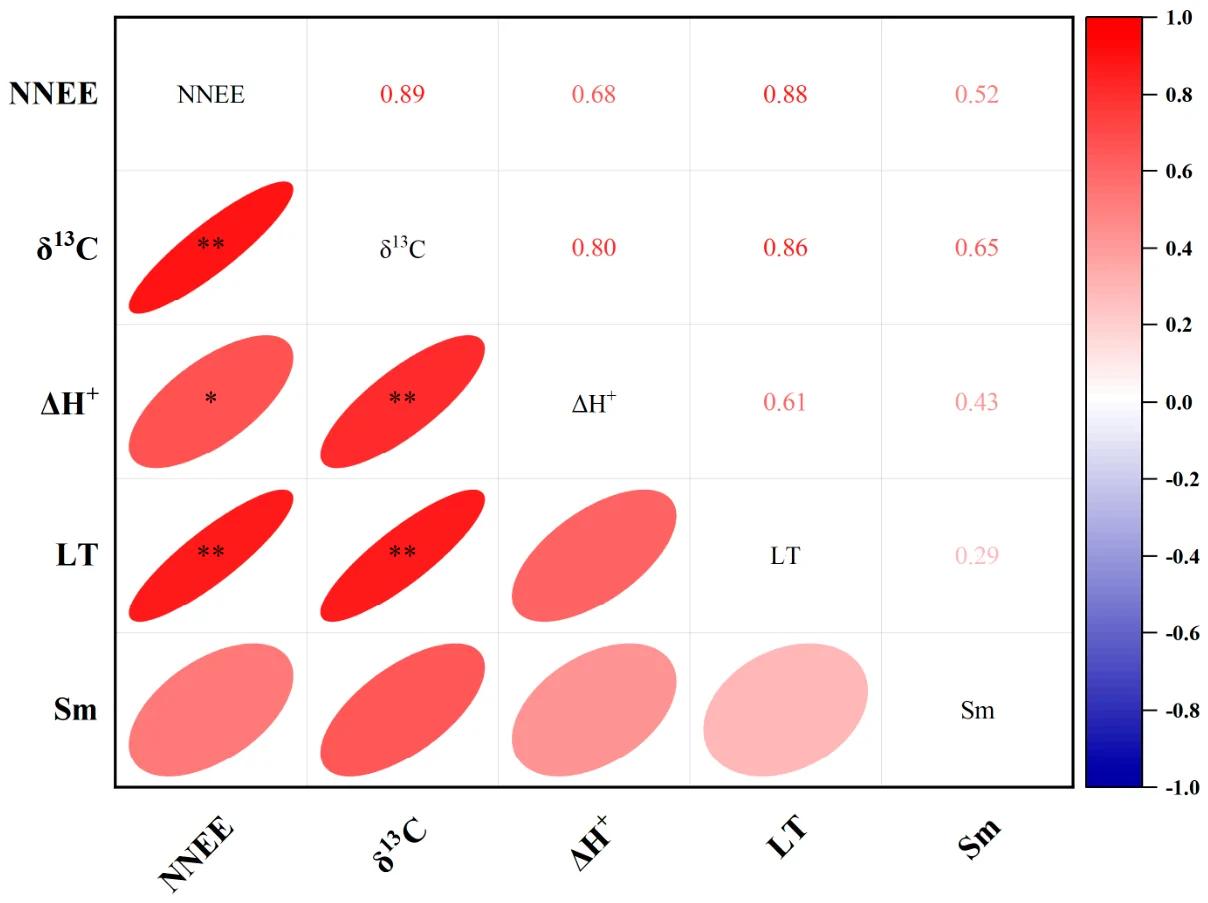
Original Pearson correlation figure for nine species (*n* = 9). NNEE retains its original label but represents the evening-to-morning sum of seven sampled rates from 18:00 to 06:00, as defined in Section 4.7; it is not an integrated nocturnal carbon balance. Color and ellipses indicate correlation direction and magnitude. * *p* < 0.05; ** *p* < 0.01 for two-sided, unadjusted tests. Coefficients and significance symbols were checked against the original matrix.

The evening-to-morning exchange index labelled NNEE in Figure 6 was positively correlated with δ^13^C (r = 0.894, *p* = 0.00113), ΔH^+^ (r = 0.676, *p* = 0.04559) and LT (r = 0.877, *p* = 0.00190). δ^13^C was positively correlated with ΔH^+^ (r = 0.805, *p* = 0.00891) and LT (r = 0.864, *p* = 0.00264). LT–ΔH^+^ did not reach the unadjusted 0.05 level (r = 0.613, *p* = 0.07926). Sm was not significantly correlated with NNEE (r = 0.521, *p* = 0.15014), δ^13^C (r = 0.649, *p* = 0.05871), ΔH^+^ (r = 0.434, *p* = 0.24320) or LT (r = 0.290, *p* = 0.44895). All correlations use nine species values; *p* values are two-sided and unadjusted for ten comparisons. These results describe trait associations rather than causation.

## 3. Discussion

### 3.1. Interpretation of Complementary Physiological Indicators

The concordance of gas exchange, isotope composition, and acidity patterns in *D. parishii*, *D. aphyllum*, and *D. anosmum* supports substantial CAM-associated assimilation under the conditions examined (Figure 1 and Figure 2; Table 1). These measurements describe complementary aspects of the same process: nocturnal carbon uptake, its contribution to tissue carbon, and overnight acid storage [23,24]. Experimental suppression of PEPC kinase in *Kalanchoë* reduced both nocturnal fixation and dawn malate accumulation, providing a mechanistic basis for this physiological association [30]. Disruption of PPC1 likewise demonstrated the central role of nocturnal carboxylation in CAM function [31]. The agreement among indicators in the present species therefore provides stronger evidence than any individual trait alone. The sampling schedule captures daytime and nighttime exchange patterns; because measurements ended at 06:00, the following 06:00–08:00 transition and a complete 24 h carbon budget remain unresolved.

*D. linawianum*, *D. moschatum*, and *D. crystallinum* combined C_3_-like isotope signatures with weak nocturnal exchange signals and variable acidity differences (Figure 1 and Figure 2). These combinations are discussed here as predominantly C_3_-associated patterns with CAM-like characteristics. Orchid surveys have documented weak nocturnal acidification in plants whose isotope composition remains within the C_3_ range [25], and controlled experiments demonstrate environmentally responsive CAM expression in epiphytic orchids [21]. In *Dendrobium*, Xia et al. [19] reported contrasting drought-associated changes in PEPC expression, providing a relevant molecular context for physiological differences among species. Water-stress experiments in *Mesembryanthemum* and reversible CAM induction in *Talinum* further show that environmental responses can involve coordinated changes in carbon fixation and gene expression [32,33,34]. These comparisons make drought–rewatering experiments a useful next step for the three species, while the current observations alone do not establish facultative CAM.

The different time scales represented by the indicators help explain their partial disagreement. Carbon isotope discrimination is influenced by diffusion and carboxylation [35], and bulk-leaf δ^13^C integrates these effects over tissue formation rather than recording one measurement day [23]. Broader surveys reveal a continuum of isotope signatures across CAM lineages [36], while field studies show that water availability can influence both isotope composition and CAM activity [37]. Thus, the relatively large acidity difference in *D. crystallinum* alongside a C_3_-like isotope signature can be interpreted as a difference between current diel acid dynamics and longer-term carbon assimilation (Figure 2). Acidity measurements also capture organic acid pools rather than atmospheric uptake alone [24]. Assessing these measurements together therefore helps characterize the extent and timing of CAM-associated behavior without requiring every indicator to assign an identical category.

Table 1 summarizes these physiological interpretations. The CAM-associated group is supported by concordant nocturnal uptake, isotope composition, and acidity differences, whereas the C_3_-associated group is characterized by predominantly daytime uptake and smaller acidity differences under the measured conditions. Species with partial concordance are retained as a separate descriptive group. This approach accommodates the diversity already reported within *Dendrobium* [16,17]. Studies of late-day fixation and light responses in CAM orchids show that the contribution of daytime carbon gain can vary substantially [22,38], so these categories describe the observed physiological state rather than an invariant response under all environments.

### 3.2. Structural Traits as Supplementary Evidence

Leaf structure can support CAM through storage capacity and the arrangement of photosynthetic tissues. Comparative work has linked tighter mesophyll packing to stronger CAM expression [39], and studies in *Clusia* connect anatomical variation with the capacity to engage CAM [40]. More recently, Gilman et al. [29] found that combinations of cell size, leaf thickness, and intercellular airspace distinguished CAM and non-CAM anatomy across a broad taxonomic sample. In our comparison, however, relatively thick leaves also occurred in C_3_-associated *D. cariniferum* (Figure 3 and Figure 4). This agrees with the finding that leaf anatomy did not consistently predict CAM function within the C_3_ + CAM hybrid *Yucca gloriosa* [28]. The difference in scale is informative: anatomical combinations can be useful across broad groups, while total leaf thickness alone may have limited discriminatory value among closely related species. The present sections provide a basis for extending the comparison to mesophyll cell dimensions and intercellular airspace.

Sm adds information on water storage relative to chlorophyll investment, and its overlap among the physiological groups suggests that succulence contributes to functions beyond nocturnal carbon fixation (Figure 5) [26,27]. Experimental work in Aizoaceae shows that succulent leaves can maintain a predominantly C_3_ strategy while buffering water loss [41]. In epiphytic orchids, cuticular protection and water stored in pseudobulbs offer additional ways to maintain water balance [15]. These findings help explain why a species can possess substantial water-storage capacity without showing strong CAM-associated exchange. Leaf thickness and Sm are therefore useful complementary traits for evaluating water relations, while gas exchange and acid dynamics provide the more direct evidence of CAM activity.

### 3.3. Associations Among Physiological and Structural Traits

The positive associations among NNEE, δ^13^C, and ΔH+ indicate coordinated variation in the physiological indicators across the nine species, whereas the weaker relationships involving Sm point to a less direct connection between succulence and carbon assimilation (Figure 6). Recent experiments provide plausible mechanisms for coordination among carbon fixation and acid dynamics. In *Portulaca*, diel regulatory studies and gene co-expression analyses connect CAM activity with the timing of carbon metabolism [42,43]. Work in the CAM orchid *Phalaenopsis* links nocturnal metabolism to the processing of starch-derived carbohydrates [44], and a starch-deficient *Kalanchoë* mutant altered the timing of stomatal CO_2_ responsiveness [45]. Comparative gene-expression studies also identify variation in the timing and recruitment of CAM-associated genes among independently evolved lineages [46]. Proteomic and phosphoproteomic evidence from *Kalanchoë* further demonstrates regulation at both transcript and protein levels [47]. These studies provide a mechanistic context for the observed covariation, while the present correlations describe species-level associations; broader sampling with phylogenetic analysis would help assess their generality.

### 3.4. Habitat Context and Practical Implications

Habitat descriptions place these physiological differences in an ecological context (Table 2) [48,49]. Epiphytic and lithophytic growth exposes orchids to intermittent water supply, and comparative orchid research has linked CAM with epiphytism and diversification [14,50]. Our species comparison identifies contrasting combinations of carbon assimilation and water-storage traits that can guide the choice of material for cultivation trials. Practical evaluation should consider the combined effects of water supply, temperature, and light: experiments with *Dendrobium* Sonia ‘Earsakul’ demonstrate distinct responses to drought, heat, and their combination [20], while work in *Phalaenopsis* shows that irradiance and CO_2_ supply influence CAM carbon gain [38]. Recent experiments with *D. nobile* and a horticultural cultivar also found different flowering and water-storage responses across drought levels [51]. Together, these studies indicate that physiological comparisons are most useful when connected to growth and reproductive performance. The present dataset provides a starting point for such tests, with native-habitat information serving as qualitative context rather than a direct measure of cultivation requirements.

## 4. Materials and Methods

### 4.1. Study Site and Plant Materials

The experiment was conducted in 2023 at the Guangxi Institute of Botany, located in the Yanshan District of Guilin City, Guangxi, China (25°01′ N, 110°17′ E). The study site is situated at an elevation of approximately 180 m and is characterized by a subtropical monsoon climate. The region experiences a mean annual temperature of 18.8 °C, with January being the coldest month, averaging 8.3 °C, and July being the hottest month, averaging 28.3 °C. Annual precipitation ranges from 1900 to 2000 mm, with over 70% occurring between April and August. The mean annual relative humidity is 78%, and the annual sunshine duration is approximately 1500 h.

Nine *Dendrobium* species were introduced from Guangxi and Yunnan and cultivated in the germplasm nursery of the Guangxi Institute of Botany. The species included *D. parishii*, *D. aphyllum*, *D. anosmum*, *D. cariniferum*, *D. gibsonii*, *D. hancockii*, *D. linawianum*, *D. moschatum*, and *D. crystallinum*, as identified by Zhongchen Xiong of the Guangxi Institute of Botany. Detailed information regarding the plant materials is provided in Table 3. The plants were grown in plastic pots (height: 18 cm; inner diameter: 16 cm) filled with tree bark, coconut fiber, and perlite in a ratio of 1:1:1. All test plants were three-year-old mature individuals. The nursery was covered with a shade net providing 30% light transmittance. Plants were routinely watered once every 5 days, but had received no irrigation for 10 days before the gas-exchange measurements in early October 2023. Leaf samples for subsequent analyses were collected after the gas-exchange measurements, except for titratable acidity, for which the specified 06:00 and 18:00 sampling times apply. Sampled leaves were mature, current-year leaves at the second or third position below the shoot apex.

### 4.2. Measurement of Diel Net CO_2_ Exchange

Net CO_2_ exchange was measured at 12 nominal two-hour sampling times from 08:00 on 7 October to 06:00 on 8 October 2023: 08:00, 10:00, 12:00, 14:00, 16:00, 18:00, 20:00, 22:00, 00:00, 02:00, 04:00, and 06:00. The schedule includes all 12 two-hour clock-time positions of a diel cycle, with 22 h between its first and last nominal observations. Healthy, fully expanded, current-year leaves at the second or third position below the shoot apex were selected. Measurements used an LI-6400XT portable photosynthesis system (LI-COR Biosciences, Lincoln, NE, USA) under ambient CO_2_ and natural light. Instrument records specify a nominal flow of 500 μmol s^−1^ and an area setting of 6 cm^2^. Three independent plants were measured per species. Repeated readings within a leaf are technical observations, not additional biological replicates. The initial 08:00 observation provides morning information but is not a measured endpoint on the following morning; complete 24 h carbon gain and the full dawn transition were not estimated.

**Figure 7:**
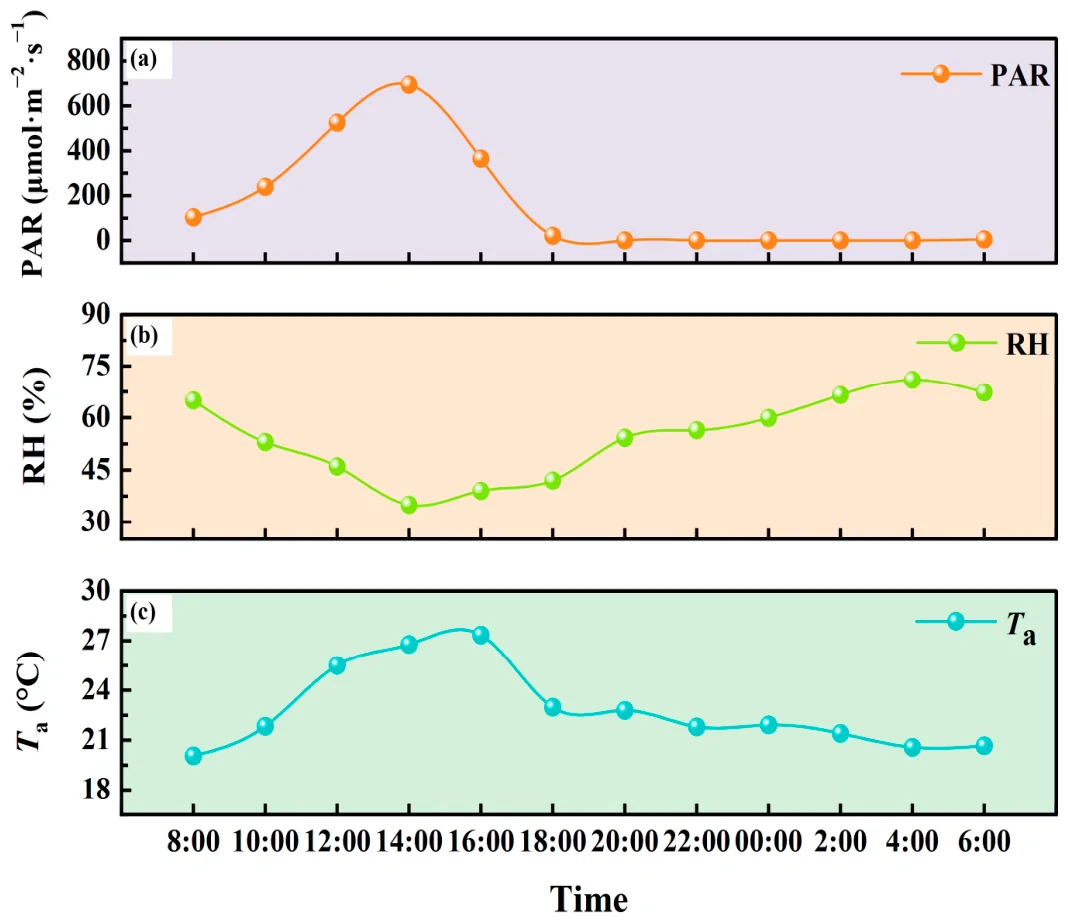
Diel variation in photosynthetically active radiation (PAR; (**a**), top), relative humidity (RH; (**b**), middle), and air temperature (Ta; (**c**), bottom) at the 12 nominal sampling times from 08:00 to 06:00 on the following morning.

PAR, air temperature and relative humidity were recorded during the measurements (Figure 7). The environmental record shows residual light at 18:00 (PAR 21.05 μmol photons m^−2^ s^−1^) and low light at the nominal 06:00 observation (4.84 μmol photons m^−2^ s^−1^). The evening-to-morning exchange summary used in the original correlation analysis therefore includes transition periods and is not a strictly dark-period measurement.

### 4.3. Measurement of Carbon Isotope Ratio

Carbon isotope ratios (δ^13^C) were determined according to Winter and Holtum [23]. Mature, current-year leaves at the second or third position below the shoot apex were collected after gas-exchange measurements, ground in liquid nitrogen, freeze-dried, and weighed for analysis. Approximately 3 mg of dried material was used per determination, as specified in the original method. Isotope ratios were measured using a Delta V Advantage isotope ratio mass spectrometer (Thermo Fisher Scientific, Bremen, Germany), with a reported analytical precision of ±0.05‰. The corrected δ^13^C column in the analytical report was used. The ratios were expressed relative to the reported PDB reference scale.

δ^13^C (‰) = 1000 × [(R_sample_/R_standard_) − 1], where R = 13C/^12^C.

Three independent plants were sampled per species for carbon isotope analysis (biological *n* = 3). Biological replication refers to independent plants, not repeated instrument determinations. Bulk-leaf δ^13^C integrates assimilation over tissue development and is not an instantaneous measure of the gas-exchange state.

### 4.4. Measurement of Titratable Acidity

Three independent plants were sampled per species for titratable acidity analysis (biological *n* = 3), with leaf samples collected at 06:00 and 18:00. Fresh tissue mass was recorded individually. Acidity was determined by endpoint titration using NaOH at 0.0927 mol L^−1^, as specified in the analytical report. The report calculates acidity as H = (V/1000) × c/(m/1000), in mol kg^−1^ FW, where V is the NaOH volume in mL, c its concentration in mol L^−1^, and m the sample fresh mass in g. Values were multiplied by 1000 to express acidity in μmol H^+^ g^−1^ FW. ΔH^+^ was defined as acidity at 06:00 minus acidity at 18:00.

### 4.5. Measurement of Leaf Anatomical Structure

Three independent plants were sampled per species, with three mature leaves per plant. Paraffin sections were prepared according to Pradhan and Bajracharya [55], observed by light microscopy using an Olympus BX53 microscope (Olympus Corporation, Tokyo, Japan), and measured in CaseViewer version 2.6 (3DHISTECH Ltd., Budapest, Hungary). Traits included upper epidermal thickness (UET), lower epidermal thickness (LET), mesophyll thickness (MT), and total leaf thickness (LT). Three sections were examined per leaf and five fields per section. For plant-level summaries, field measurements were averaged within sections, section means within leaves, and leaf means within plants; species means and between-plant variability were then calculated from the three plant means (biological *n* = 3). The 135 fields per species are nested subsamples, not 135 independent biological replicates. For the updated component-based comparison, LT = UET + MT + LET.

### 4.6. Measurement of the Mesophyll Succulence Index

The mesophyll succulence index (Sm) was evaluated following Kluge and Ting [27] and Males [26] as water mass per unit chlorophyll mass. Leaf water content and chlorophyll content were expressed on the same fresh-mass basis. Chlorophyll content was expressed in mg g^−1^ FW; the previous area-based unit, mg dm^−2^, was a reporting error.

Sm = [(FW − DW)/FW]/ChlFW = (FW − DW)/(FW × ChlFW), where FW and DW are fresh and dry masses in g and ChlFW is total chlorophyll content in mg g^−1^ FW. Sm is therefore expressed in g water mg^−1^ chlorophyll. Water fraction and chlorophyll concentration must represent the same plant and comparable leaf material.

For each species, a total of six to ten mature leaves were collected from three independent plants, weighed fresh (FW), and dried at 75 °C to constant mass (DW). Water mass is FW − DW, and the fresh-mass water fraction is (FW − DW)/FW. This fraction is not relative water content, which requires turgid mass.

Water mass (g) = FW − DW; fresh-mass water fraction = (FW − DW)/FW.

A 0.2 g fresh leaf sample was made up to 25 mL with 95% ethanol and extracted for 24 h in darkness. Absorbance at 665 and 649 nm was measured using a UV-Vis spectrophotometer (PerkinElmer, Shelton, CT, USA). Extract concentrations were calculated as Ca = 13.95 × A_665_ − 6.88 × A_649_ and Cb = 24.96 × A_649_ − 7.32 × A_665_, in mg L^−1^. Total chlorophyll content was ChlFW = (Ca + Cb) × V × N/(1000 × m), where V is extract volume in mL, N the dilution factor and m the extracted fresh mass in g. ChlFW is in mg g^−1^ FW. Three replicates were used per species.

### 4.7. Data Analyses

Data were organized in Microsoft Excel 2019, and the original two-sided Pearson correlations were calculated in SPSS Statistics 27.0. To retain the original figure and analysis, the variable labelled NNEE is defined here as an evening-to-morning exchange index: the sum of seven species-level net CO_2_ exchange rates at nominal 18:00, 20:00, 22:00, 00:00, 02:00, 04:00 and 06:00. This is a sum of sampled rates, not a time-integrated carbon balance or a strictly dark-period mean. Dividing the index by seven would yield the corresponding arithmetic mean without changing Pearson r. Each species contributes one value per trait (*n* = 9 species). The ten pairwise coefficients and two-sided *p* values were checked against the original species-level matrix. The *p* values are unadjusted for multiple comparisons and interpreted descriptively. Phylogenetic relationships were not modelled, so correlations are not interpreted as causal or independent evolutionary relationships.

## 5. Conclusions

The nine *Dendrobium* species showed contrasting physiological patterns under the conditions examined. *D. parishii*, *D. aphyllum*, and *D. anosmum* exhibited concordant evidence consistent with substantial CAM-associated assimilation, whereas *D. cariniferum*, *D. gibsonii*, and *D. hancockii* showed predominantly C_3_-associated patterns. *D. linawianum*, *D. moschatum*, and *D. crystallinum* showed C_3_-like isotope signatures with reported CAM-like characteristics requiring validation. These observations do not establish facultative CAM, obligate pathway status, or environmental induction. Leaf thickness and Sm provide supplementary information and cannot independently identify pathways. Exploratory correlations and qualitative habitat comparisons generate hypotheses for further testing. This comparison provides a basis for selecting species and traits for cultivation and conservation research. Future studies with larger sample sizes and repeated diel measurements would help assess the generality of these patterns.

## Figures and Tables

**Table 1:** Reported species-level physiological values and provisional interpretation. NNEE is the evening-to-morning sum of seven sampled exchange rates (μmol CO_2_ m^−2^ s^−1^), not an integrated flux; δ^13^C is in ‰ and ΔH^+^ in μmol H^+^ g^−1^ FW. The author confirms three independent plants in the biological design. Categories are descriptive and not universal diagnostic thresholds.

Species	NNEE	δ^13^C	ΔH^+^	Provisional Interpretation
*D. parishii*	9.40076	−14.382	46.373	CAM-associated evidence
*D. aphyllum*	7.96467	−15.476	143.544	CAM-associated evidence
*D. anosmum*	4.21217	−15.953	112.332	CAM-associated evidence
*D. cariniferum*	−1.10686	−29.762	18.362	C_3_-associated evidence
*D. gibsonii*	−2.09557	−26.434	10.554	C_3_-associated evidence
*D. hancockii*	−1.06556	−32.584	1.088	C_3_-associated evidence
*D. linawianum*	0.68070	−27.370	18.573	C_3_-associated with CAM-like characteristics
*D. moschatum*	2.24060	−28.453	6.435	C_3_-associated with CAM-like characteristics
*D. crystallinum*	−0.12981	−28.031	47.355	C_3_-associated with CAM-like characteristics

**Table 2:** Natural habitat distribution of nine *Dendrobium* species.

Species	Native Distribution	Habitat Type (as Documented in the Literature)	References
*D. parishii*	Northeast India, Myanmar, Thailand, Laos, Vietnam, China (Yunnan)	Epiphytic on trees in montane evergreen forests or on rocks; elevation 250–1200 m	[48,49]
*D. aphyllum*	Nepal, Bhutan, India, Myanmar, Thailand, Laos, Vietnam, China (Yunnan)	Epiphytic or lithophytic in tropical moist forests; elevation 200–1500 m	[48,49]
*D. anosmum*	Philippines, Malaysia, Laos, Vietnam, Myanmar, China (Yunnan)	Epiphytic in lowland to montane forests, often near riverbanks or humid valleys	[48,52]
*D. cariniferum*	China (Yunnan), Myanmar, Northeast India	Epiphytic in humid montane forests; elevation 1200–2000 m	[48,49]
*D. gibsonii*	China (Yunnan, Guangxi), India, Myanmar	Epiphytic or lithophytic on cliffs, rocks or forest trunks; elevation 800–1500 m	[48,49]
*D. hancockii*	China (Yunnan, Guizhou, Guangxi), Vietnam, Northern Thailand	Epiphytic or lithophytic in montane forests; elevation 700–1500 m	[49,53]
*D. linawianum*	Taiwan, China (Guangdong)	Epiphytic in montane evergreen forests; elevation 700–1400 m	[48,49]
*D. moschatum*	Northeast India, Nepal, Bhutan, Myanmar, China (Yunnan)	Epiphytic in cliffs or montane moist forests; elevation 1000–1800 m	[48,54]
*D. crystallinum*	Thailand, Myanmar, Laos, Vietnam, China (Yunnan, Guizhou, Hainan)	Epiphytic or lithophytic in montane forests with seasonal moisture variation; elevation 700–1700 m	[48,49]

**Table 3:** Experimental materials.

Species	Determiner	Determining Institution	Site of Introduction
*D. parishii*	Zhongchen Xiong	Guangxi Institute of Botany	Baoshan, Yunnan
*D. aphyllum*	Zhongchen Xiong	Guangxi Institute of Botany	Pu’er, Yunnan
*D. anosmum*	Zhongchen Xiong	Guangxi Institute of Botany	Longzhou, Guangxi
*D. cariniferum*	Zhongchen Xiong	Guangxi Institute of Botany	Tian’e, Guangxi
*D. gibsonii*	Zhongchen Xiong	Guangxi Institute of Botany	Baoshan, Yunnan
*D. hancockii*	Zhongchen Xiong	Guangxi Institute of Botany	Baise, Guangxi
*D. linawianum*	Zhongchen Xiong	Guangxi Institute of Botany	Jinxiu, Guangxi
*D. moschatum*	Zhongchen Xiong	Guangxi Institute of Botany	Jinghong, Yunnan
*D. crystallinum*	Zhongchen Xiong	Guangxi Institute of Botany	Tian’e, Guangxi

## Data Availability

All data generated or analyzed during this study are included in this published article.

## References

[1] Blankenship R.E. (2021). Molecular Mechanisms of Photosynthesis.

[2] Nelson N., Yocum C.F. (2006). Structure and function of photosystems I and II. Annu. Rev. Plant Biol..

[3] Ehleringer J.R., Monson R.K. (1993). Evolutionary and ecological aspects of photosynthetic pathway variation. Annu. Rev. Ecol. Syst..

[4] Sage R.F. (2004). The evolution of C_4_ photosynthesis. New Phytol..

[5] Sage R.F., Zhu X.-G. (2011). Exploiting the engine of C_4_ photosynthesis. J. Exp. Bot..

[6] Lüttge U. (2004). Ecophysiology of crassulacean acid metabolism (CAM). Ann. Bot..

[7] Winter K., Smith J.A.C., Winter K., Smith J.A.C. (1996). Crassulacean acid metabolism: Current status and perspectives. Crassulacean Acid Metabolism: Biochemistry, Ecophysiology and Evolution.

[8] Borland A.M., Griffiths H., Hartwell J., Smith J.A.C. (2009). Exploiting the potential of plants with crassulacean acid metabolism for bioenergy production on marginal lands. J. Exp. Bot..

[9] Winter K., Holtum J.A. (2014). Facultative crassulacean acid metabolism (CAM) plants: Powerful tools for unravelling the functional elements of CAM photosynthesis. J. Exp. Bot..

[10] Osmond C.B. (1978). Crassulacean acid metabolism: A curiosity in context. Annu. Rev. Plant Physiol..

[11] Ting I.P. (1985). Crassulacean acid metabolism. Annu. Rev. Plant Physiol..

[12] Hou B., Luo J., Zhang Y., Niu Z., Xue Q., Ding X. (2017). Iteration expansion and regional evolution: Phylogeography of *Dendrobium officinale* and four related taxa in southern China. Sci. Rep..

[13] Cheng J., Dang P., Zhao Z., Yuan L., Zhou Z., Wolf D., Luo Y. (2019). An assessment of the Chinese medicinal *Dendrobium* industry: Supply, demand and sustainability. J. Ethnopharmacol..

[14] Zhang S., Yang Y., Li J., Qin J., Zhang W., Huang W., Hu H. (2018). Physiological diversity of orchids. Plant Divers..

[15] Yang S.-J., Sun M., Yang Q.-Y., Ma R.-Y., Zhang J.-L., Zhang S.-B. (2016). Two strategies by epiphytic orchids for maintaining water balance: Thick cuticles in leaves and water storage in pseudobulbs. AoB Plants.

[16] Zhang Z., He D., Niu G., Gao R. (2014). Concomitant CAM and C_3_ photosynthetic pathways in *Dendrobium officinale* plants. J. Am. Soc. Hortic. Sci..

[17] Qiu S., Sultana S., Liu Z.D., Yin L.Y., Wang C.Y. (2015). Identification of obligate C_3_ photosynthesis in *Dendrobium*. Photosynthetica.

[18] Xue Q., Yang J., Yu W., Wang H., Hou Z., Li C., Xue Q., Liu W., Ding X., Niu Z. (2023). The climate changes promoted the chloroplast genomic evolution of *Dendrobium* orchids among multiple photosynthetic pathways. BMC Plant Biol..

[19] Xia K., Wu Q., Yang Y., Liu Q., Wang Z., Zhao Z., Li J., He J., Chai S., Qiu S. (2024). Drought Stress Induced Different Response Mechanisms in Three *Dendrobium* Species under Different Photosynthetic Pathways. Int. J. Mol. Sci..

[20] Taticharoen T., Matsumoto S., Chutteang C., Srion K., Malumpong C., Abdullakasim S. (2023). Response and acclimatization of a CAM orchid, *Dendrobium* Sonia ‘Earsakul’ to drought, heat, and combined drought and heat stress. Sci. Hortic..

[21] Tay S., He J., Yam T.W. (2019). CAM plasticity in epiphytic tropical orchid species responding to environmental stress. Bot. Stud..

[22] van Tongerlo E., Trouwborst G., Hogewoning S.W., van Ieperen W., Dieleman J.A., Marcelis L.F. (2021). Crassulacean acid metabolism species differ in the contribution of C_3_ and C_4_ carboxylation to end-of-day CO_2_ fixation. Physiol. Plant..

[23] Winter K., Holtum J.A. (2002). How closely do the δ13C values of crassulacean acid metabolism plants reflect the proportion of CO_2_ fixed during day and night?. Plant Physiol..

[24] Winter K., Smith J.A.C. (2022). CAM photosynthesis: The acid test. New Phytol..

[25] Silvera K., Santiago L.S., Winter K. (2005). Distribution of crassulacean acid metabolism in orchids of Panama: Evidence of selection for weak and strong modes. Funct. Plant Biol..

[26] Males J. (2017). Secrets of succulence. J. Exp. Bot..

[27] Kluge M., Ting I.P. (1978). Crassulacean Acid Metabolism: Analysis of an Ecological Adaptation.

[28] Heyduk K., Ray J.N., Leebens-Mack J. (2021). Leaf anatomy is not correlated to CAM function in a C_3_ + CAM hybrid species, *Yucca gloriosa*. Ann. Bot..

[29] Gilman I.S., Heyduk K., Maya-Lastra C., Hancock L.P., Edwards E.J. (2024). Predicting photosynthetic pathway from anatomy using machine learning. New Phytol..

[30] Boxall S.F., Dever L.V., Kneřová J., Gould P.D., Hartwell J. (2017). Phosphorylation of phosphoenolpyruvate carboxylase is essential for maximal and sustained dark CO_2_ fixation and core circadian clock operation in the obligate crassulacean acid metabolism species *Kalanchoë fedtschenkoi*. Plant Cell.

[31] Boxall S.F., Kadu N., Dever L.V., Kneřová J., Waller J.L., Gould P.J.D., Hartwell J. (2020). *Kalanchoë* PPC1 is essential for crassulacean acid metabolism and the regulation of core circadian clock and guard cell signaling genes. Plant Cell.

[32] Eastmond P.J., Ross J.D. (1997). Evidence that the induction of crassulacean acid metabolism by water stress in *Mesembryanthemum crystallinum* (L.) involves root signalling. Plant Cell Environ..

[33] Winter K., Holtum J.A.M. (2007). Environment or development? Lifetime net CO_2_ exchange and control of the expression of crassulacean acid metabolism in *Mesembryanthemum crystallinum*. Plant Physiol..

[34] Brilhaus D., Bräutigam A., Mettler-Altmann T., Winter K., Weber A.P.M. (2016). Reversible burst of transcriptional changes during induction of crassulacean acid metabolism in *Talinum triangulare*. Plant Physiol..

[35] Farquhar G.D., O’Leary M.H., Berry J.A. (1982). On the relationship between carbon isotope discrimination and the intercellular carbon dioxide concentration in leaves. Aust. J. Plant Physiol..

[36] Messerschmid T.F., Wehling J., Bobon N., Kahmen A., Klak C., Los J.A., Nelson D.B., dos Santos P., de Vos J.M., Kadereit G. (2021). Carbon isotope composition of plant photosynthetic tissues reflects a crassulacean acid metabolism (CAM) continuum in the majority of CAM lineages. Perspect. Plant Ecol. Evol. Syst..

[37] Ricalde M.F., Andrade J.L., Durán R., Dupuy J.M., Simá J.L., Us-Santamaría R., Santiago L.S. (2010). Environmental regulation of carbon isotope composition and crassulacean acid metabolism in three plant communities along a water availability gradient. Oecologia.

[38] Hogewoning S.W., van den Boogaart S.A., van Tongerlo E., Trouwborst G. (2021). CAM physiology and carbon gain of the orchid *Phalaenopsis* in response to light intensity, light integral and CO_2_. Plant Cell Environ..

[39] Nelson E.A., Sage R.F. (2008). Functional constraints of CAM leaf anatomy: Tight cell packing is associated with increased CAM function across a gradient of CAM expression. J. Exp. Bot..

[40] Barrera Zambrano V.A., Lawson T., Olmos E., Fernández-García N., Borland A.M. (2014). Leaf anatomical traits which accommodate the facultative engagement of crassulacean acid metabolism in tropical trees of the genus *Clusia*. J. Exp. Bot..

[41] Ripley B.S., Abraham T., Klak C., Cramer M.D. (2013). How succulent leaves of Aizoaceae avoid mesophyll conductance limitations of photosynthesis and survive drought. J. Exp. Bot..

[42] Ferrari R.C., Kawabata A.B., Ferreira S.S., Hartwell J., Freschi L. (2022). A matter of time: Regulatory events behind the synchronization of C_4_ and crassulacean acid metabolism in *Portulaca oleracea*. J. Exp. Bot..

[43] Gilman I.S., Moreno-Villena J.J., Lewis Z.R., Goolsby E.W., Edwards E.J. (2022). Gene co-expression reveals the modularity and integration of C_4_ and CAM in *Portulaca*. Plant Physiol..

[44] Ceusters N., Frans M., Van den Ende W., Ceusters J. (2019). Maltose processing and not β-amylase activity curtails hydrolytic starch degradation in the CAM orchid *Phalaenopsis*. Front. Plant Sci..

[45] Hurtado-Castano N., Atkins E., Barnes J., Boxall S.F., Dever L.V., Kneřová J., Hartwell J., Cushman J.C., Borland A.M. (2023). The starch-deficient plastidic PHOSPHOGLUCOMUTASE mutant of the constitutive crassulacean acid metabolism (CAM) species *Kalanchoë fedtschenkoi* impacts diel regulation and timing of stomatal CO_2_ responsiveness. Ann. Bot..

[46] Heyduk K., McAssey E.V., Leebens-Mack J. (2022). Differential timing of gene expression and recruitment in independent origins of CAM in the Agavoideae (Asparagaceae). New Phytol..

[47] Schiller K., Janshoff S., Zenker S., Viehöver P., Hartwell J., Eirich J., Finkemeier I., Bräutigam A. (2025). Regulation of Crassulacean acid metabolism at the protein level in *Kalanchoë laxiflora*. Plant Physiol..

[48] Plants of the World Online. Royal Botanic Gardens, Kew. https://powo.science.kew.org.

[49] Flora of China. http://www.efloras.org/flora_page.aspx?flora_id=2.

[50] Silvera K., Santiago L.S., Cushman J.C., Winter K. (2009). Crassulacean acid metabolism and epiphytism linked to adaptive radiations in the Orchidaceae. Plant Physiol..

[51] Zhou Y., Meng Y.-Y., Long Q.-Y., Li J.-W. (2025). Plasticity in *Dendrobium* floral structure and physiological response under different drought stress levels. Sci. Hortic..

[52] New Guinea Orchids. https://www.orchidsnewguinea.com.

[53] Smithsonian National Museum of Natural History. https://naturalhistory.si.edu.

[54] Useful Tropical Plants. http://tropical.theferns.info.

[55] Pradhan B.M., Bajracharya D.M. (2020). Anatomical study of *Dendrobium* (Orchidaceae) of Nepal. Ann. Plant Sci..

